# Task-Oriented Multi-View Acquisition Planning for Visual Inspection of Panel Furniture

**DOI:** 10.3390/s26196188

**Published:** 2026-09-29

**Authors:** Wenxu Luo, Zhuo Wang, Sundong Mo, Yuanyuan Wang

**Affiliations:** College of Control and Information Engineering, Northeast Forestry University, Harbin 150040, China

**Keywords:** furniture visual inspection, inspection-surface prioritization, viewpoint planning, coverage path planning, ant colony optimization

## Abstract

Automated multi-view acquisition is widely used in industrial production but remains underexplored for furniture inspection. This study proposes a task-oriented multi-view acquisition planning method for panel furniture. The method constructs a hierarchical inspection-surface model from furniture geometry and inspection requirements, evaluates target visibility by one-sided triangle-mesh ray casting, and iteratively refines the candidate pool based on coverage residuals. Viewpoints for must-inspect surfaces are selected using a quality-aware marginal score, with their number determined by comparing local pose replacement with continued viewpoint addition. Supplementary viewpoints for optional surfaces are ranked by coverage gain, and their number is determined using the L-method. Visit sequences are optimized using ant colony optimization and 2-opt under a hard keep-out-zone constraint. For four furniture models, 19, 23, 30, and 72 viewpoints achieved must-inspect-surface coverage rates of 100.00%, 100.00%, 99.86%, and 98.16%, respectively. For matched numbers of must-inspect-surface viewpoints, 25° effective coverage exceeded the 20-run means of genetic algorithm and binary particle swarm optimization baselines by 1.19–13.16 and 20.09–36.72 percentage points, respectively. Under fixed total viewpoint counts, local pose replacement increased must-inspect-surface coverage by 0.10–1.72 percentage points. Across 20 paired trials per model, keep-out-zone-constrained sequencing produced zero intersecting edges while increasing path length by 1.33–6.91%. These results demonstrate the effectiveness of the proposed method for front-end multi-view acquisition planning in panel furniture inspection.

## 1. Introduction

### 1.1. Research Background

Machine vision is widely used in furniture manufacturing for surface quality inspection, dimensional measurement and positioning, and process monitoring. Relatively mature applications have been established in standardized operations such as panel inspection, edge banding, and hole-position detection [1]. Panel furniture represents an important segment of the wooden-furniture market, accounting for approximately 58.2% of China’s wooden-furniture market according to a recent study [2]. It is composed mainly of panels with regular geometries, and its component forms and manufacturing processes are highly standardized, providing favorable conditions for machine-vision applications. Existing research and industrial applications have therefore focused mainly on upstream processes such as panel processing, edge banding, and drilling. In these operations, the inspected object is typically a single panel or local planar surface, resulting in relatively fixed viewing directions and well-defined camera arrangements and inspection workspaces. By contrast, appearance inspection of assembled and coated panel furniture still relies largely on manual visual examination, which is affected by operator experience, visual fatigue, illumination conditions, and viewing angles. This reliance can lead to inconsistent judgments, low inspection efficiency, and missed defects in localized areas.

Unlike the two-dimensional inspection of individual panels during upstream manufacturing, the inspection of finished panel furniture involves multiple components, including panels, legs, beams, backrests, and armrests, with the surfaces to be inspected oriented in different directions. Furniture geometry and local occlusions lead to substantial differences among regions in feasible viewing directions, working distances, and available camera placement space. Even geometrically visible surfaces may fail to yield images that meet inspection requirements because of excessive incidence angles, unsuitable working distances, or occlusion by nearby components. For objects with known three-dimensional models, viewpoint planning generally accounts for constraints on the field of view, working distance, viewing direction, and visibility [3,4]. Inspection priorities also vary across surfaces. Primary appearance surfaces require prioritized and complete acquisition, whereas interior and underside surfaces have supplementary inspection value and should not be subject to the same coverage requirements. Extending existing two-dimensional visual inspection techniques to finished panel furniture therefore requires automated multi-view image acquisition under three-dimensional structural constraints.

To address these image-acquisition requirements, this study focuses on offline multi-view planning for furniture with known geometric models. Existing studies commonly adopt a staged planning framework comprising candidate-viewpoint generation, viewpoint selection, and visit-sequence optimization [5,6]. Subject to constraints on the field of view, working distance, incidence angle, and visibility, the planned viewpoints should first ensure complete coverage of primary appearance surfaces while also covering supplementary inspection regions such as interior and underside surfaces. The selected viewpoints must also be arranged in a suitable visit sequence so that straight-line connections between consecutive viewpoints do not intersect predefined keep-out zones. Accordingly, this study addresses three interrelated components: inspection-surface prioritization, candidate-viewpoint generation and selection, and keep-out-zone-constrained visit sequencing.

### 1.2. Related Work

Research on machine vision in furniture manufacturing has primarily focused on two-dimensional image-based inspection of regular objects such as panels, wood veneers, and edge-banded components. Relevant methods have evolved from handcrafted features based on texture, color, and edges to deep-learning approaches based primarily on convolutional neural networks and object-detection networks [1]. Chen et al. [7] developed a deep-learning detection pipeline for defects such as open knots, holes, and cracks in edge-glued wood panels. Ge et al. [8] employed a multiscale DETR model to detect surface defects in wood veneers. Wang et al. [9] improved the multiscale feature-extraction and small-object-detection capabilities of YOLOv8, thereby increasing the accuracy of wood surface defect detection. These studies have improved defect detection on regular wooden surfaces and provided a technical foundation for automated furniture quality inspection. However, because these methods generally use previously acquired two-dimensional images as inputs, image acquisition for assembled furniture surfaces oriented in different directions remains underexplored.

In contrast to two-dimensional image-based defect recognition, automated visual acquisition for complex three-dimensional objects has been studied extensively in applications such as the inspection of industrial parts, curved panels, and aerospace components. Chen and Li [10] planned visual-sensor poses and corresponding visit paths based on known geometric models. Chatterjee and Issac [11] investigated viewpoint planning and multi-view image stitching for curved panels. Nandagopal et al. [12] developed a surface-inspection framework for complex parts that integrates mesh partitioning, ray-based viewpoint generation, and robotic acquisition. Overall, existing studies have focused mainly on industrial parts, free-form surfaces, and complex engineering components. By comparison, automated visual acquisition for the differently oriented surfaces of assembled furniture has received limited attention.

In automated visual acquisition, model-driven viewpoint planning generates and filters candidate camera poses so that target surfaces meet visibility and observation-quality requirements. Trucco et al. [3] formulated sensor placement as a combinatorial optimization problem subject to model-based constraints. Bonaventura et al. [4] systematically reviewed viewpoint-quality evaluation methods for polygonal models, and Scott [13] further summarized the relationships among candidate generation, visibility evaluation, and viewpoint selection. Subsequently, Mosbach et al. [14] performed nonuniform candidate sampling based on curvature and edge features, assigning higher candidate densities to geometrically complex regions. Gospodnetić et al. [15] compared several generate-and-test viewpoint-placement strategies and showed that candidate-generation methods, sensor models, and evaluation metrics jointly affect planning results. Chatterjee and Issac [16] partitioned panel surfaces using geodesics and generated corresponding camera viewpoints and coverage paths. These studies indicate that the spatial distribution of candidate poses and the accuracy of visibility evaluation jointly determine the upper bound on coverage attainable through subsequent viewpoint selection.

In viewpoint selection, the problem is commonly formulated as a set-cover problem, next-best-view planning, or discrete optimization under combinatorial constraints. Lauri et al. [17] formulated multi-sensor next-best-view planning as a matroid-constrained submodular maximization problem to balance information gain and selection constraints under a limited budget. Zhao et al. [5] proposed a closely related workflow for the multi-robot inspection planning of large free-form surfaces. Their method first generates a redundant viewpoint set from product geometry, filters viewpoints subject to complete-coverage and measurement-uncertainty constraints, and then performs multi-robot task allocation and visit-sequence planning. This process reflects a hierarchical optimization framework of “viewpoint sampling–viewpoint selection–path planning.” Glorieux et al. [18] reduced the number of ineffective candidates in free-form surface inspection through targeted viewpoint sampling. Liu et al. [19] further incorporated measurement uncertainty into viewpoint sampling and coverage planning to balance the number of viewpoints against measurement quality. Existing methods have gradually shifted from pursuing geometric coverage alone toward jointly considering measurement quality and inspection efficiency. However, they mainly address dimensional inspection of free-form surfaces, where surface importance is typically characterized by geometric features or measurement uncertainty, with limited differentiation among the inspection priorities of primary appearance, interior, and underside surfaces.

Beyond viewpoint selection, coverage path planning considers how to visit all inspection targets or selected viewpoints while minimizing travel cost. Galceran and Carreras [20] systematically reviewed the main modeling approaches for robotic coverage path planning. Englot and Hover [21] constructed a redundant sampling graph and searched for coverage paths for the inspection of complex structures. Bircher et al. [6,22] alternated between viewpoint resampling and tour optimization, thereby coupling viewpoint selection with path-length optimization. Jing et al. [23] addressed local coverage and path connectivity in visual inspection using path primitives and a primitive coverage graph. For industrial nondestructive testing, Olivieri et al. [24] and Felsner et al. [25] investigated path planning for eddy-current and ultrasonic inspection of complex aerospace components, respectively. Zhao et al. [26] repaired unsafe paths through interference checking in on-machine measurement of free-form surfaces. These studies provide an important foundation for the inspection of complex structures. However, some methods focus on continuous scanning trajectories, whereas others optimize the visit sequence independently after the viewpoints have been determined. For discrete-viewpoint acquisition of furniture, satisfying observation constraints at individual viewpoints does not guarantee feasible connections between consecutive viewpoints; a high-coverage viewpoint set may still contain edges that intersect keep-out zones.

Building on these studies, recent viewpoint-planning research has increasingly considered observation quality, task information, and execution constraints in an integrated manner. Kong et al. [27] jointly optimized station and viewpoint coverage for mobile visual inspection. Vutetakis and Xiao [28] and Li et al. [29] investigated viewpoint planning from the perspectives of non-myopic active perception and online scene reconstruction, respectively. For specific inspection tasks, Liu et al. [30] incorporated structural semantics and contextual information, Li et al. [31] used defect-sensitive regions to guide blisk viewpoint planning, and Pi et al. [32] combined curvature-adaptive viewpoint generation with robot path optimization for large-scale free-form surfaces. These studies have broadened the consideration of observation quality, task information, and execution constraints in viewpoint planning, reflecting a gradual shift from geometric coverage alone toward multi-factor coordinated planning.

In summary, existing studies have established a substantial technical foundation for two-dimensional defect recognition, model-driven viewpoint generation, quality-aware viewpoint selection, and inspection path planning. However, several limitations remain when these techniques are applied to panel furniture. First, existing visual-inspection methods for panel furniture generally use previously acquired two-dimensional images as inputs, with limited attention to automated acquisition planning for the differently oriented surfaces of coated or assembled furniture. Second, uniform coverage requirements are often applied to different inspection surfaces, making it difficult to distinguish the inspection priorities of primary appearance surfaces and supplementary inspection regions. Single-pass candidate generation is also insufficient for adequately handling interior, boundary, and occluded regions. Finally, if the visit sequence is optimized solely according to distance after viewpoint selection, without additional constraints, the resulting sequence may still contain connections that intersect keep-out zones. To address these issues, this study develops a multi-view acquisition planning workflow comprising inspection-surface prioritization, iterative candidate-pool refinement, two-stage viewpoint selection, and keep-out-zone-constrained visit sequencing.

### 1.3. Overview and Contributions

**Figure 1:**
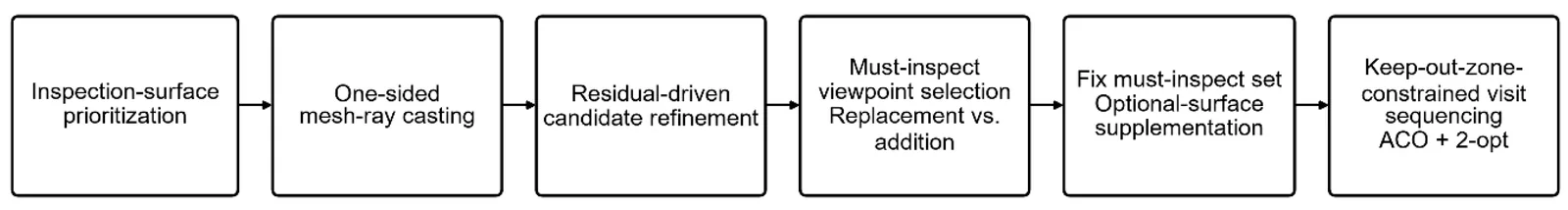
Proposed multi-view acquisition planning framework.

Figure 1 presents the proposed multi-view acquisition planning framework for the visual inspection of panel furniture. The framework takes a furniture STL model and a predefined inspection task as inputs. The inspection task specifies the inspection priorities and corresponding observation requirements of different surfaces, based on which the surfaces are classified as must-inspect, optional, or ignored. The framework then sequentially performs inspection-surface prioritization, candidate-space construction, two-stage viewpoint selection, and keep-out-zone-constrained visit sequencing, and finally outputs a viewpoint set satisfying the observation constraints together with the corresponding visit sequence.

The main contributions of this study are as follows:

1. A task-oriented inspection-surface prioritization method is proposed for panel furniture. It determines which surfaces are externally exposed, assigns surfaces to task categories such as must-inspect and optional, and generates area-weighted target points, thereby producing a target model with explicit inspection priorities for subsequent viewpoint planning.
2. A residual-driven candidate-pool refinement and quality-aware viewpoint selection method is proposed. Targeted candidate supplementation expands effective coverage in constrained regions, while local pose replacement repairs residual coverage gaps. The number of supplementary viewpoints for optional surfaces is determined using the L-method [33].
3. A keep-out-zone-constrained viewpoint sequencing method is proposed. Viewpoint-connection edges intersecting the predefined keep-out zone are excluded during ACO construction, and the same edge-feasibility constraint is enforced during 2-opt refinement.

## 2. Problem Formulation and Overall Framework

### 2.1. Inspection Task

This study aims to plan camera viewpoints that prioritize coverage of must-inspect surfaces while providing supplementary acquisition of optional surfaces, subject to observation constraints. The planning process also seeks to reduce redundant viewpoints and improve acquisition efficiency. The problem is formulated as offline multi-view planning for a given furniture model, camera parameters, and planning coordinate frame. The outputs are a viewpoint set *S* satisfying the observation constraints and its open-path visit sequence *π*.

To represent different inspection priorities, the externally exposed triangular faces are classified into the must-inspect set *F_m_*, *L* optional-surface classes, denoted by *F_o_*_,*l*_ for *l* = 1,…, *L*, and the ignored set *F_g_*. These labels jointly specify the inspection priority of each exposed surface under the current inspection task. Must-inspect surfaces require prioritized and complete acquisition. Optional surfaces are included in supplementary acquisition after the must-inspect requirements have been satisfied, whereas ignored surfaces are excluded from the current planning process.

An area-weighted target-point set *P* is generated on the must-inspect and optional surfaces. The must-inspect target-point set is denoted by *P_m_*, and the target-point set of class *l* is denoted by *P_l_*. The position, unit normal, and representative area weight of target point *i* are denoted by *x_i_*, *n_i_*, and *w_i_*, respectively. These area weights allow subsequent coverage rates to be calculated according to the actual surface area rather than the number of sampled points.

A candidate viewpoint *v* = (*c_v_*,*t_v_*) is represented by its camera center *c_v_* and look-at point *t_v_*, and must satisfy the constraints imposed by the allowable workspace and furniture keep-out zones. A target point *i* is considered visible from candidate viewpoint *v* only if it simultaneously lies within both the field of view and the valid observation-distance range, faces the camera, satisfies the incidence-angle constraint, and has an unobstructed line of sight. The visibility indicator is defined as *a_iv_* = 1 when these conditions are satisfied and *a_iv_* = 0 otherwise. The incidence angle, projection quality, and observation distance of target point *i* at viewpoint *v* are denoted by *θ_iv_*, *q_iv_*, and *d_iv_*, respectively. This visibility relationship is used in the subsequent evaluation of surface coverage and observation quality.

### 2.2. Planning Objectives

The planning process sequentially determines the viewpoint set for must-inspect surfaces, the supplementary set for optional surfaces, and the visit sequence. The must-inspect stage outputs the viewpoint set $S_{m}^{*}$, whose cardinality is given by Equation (1):

(1) $$K_{m}=\left|S_{m}^{⋆}\right|$$

The optional-surface stage outputs the supplementary set *S_o_*, yielding the final viewpoint set given by Equation (2):

(2) $$S=S_{m}^{⋆}\cup S_{o}$$

Once the viewpoint set *S* has been determined, the visit-sequencing stage optimizes only its open-path visit sequence *π*, without changing the viewpoint positions or their number. For a given viewpoint set *S*, the area-weighted geometric coverage rate of inspection-surface category *l* and its effective coverage rate under an incidence-angle threshold *τ* are defined by Equations (3) and (4), respectively:

(3) $$C_{l}\left(S\right)=\frac{\sum_{i\in P_{l}}w_{i}\;I\;\left[\exists v\in S:a_{\mathrm{iv}}=1\right]}{\sum_{i\in P_{l}}w_{i}}$$

(4) $$C_{l,\tau }\left(S\right)=\frac{\sum_{i\in P_{l}}w_{i}\;I\;\left[\exists v\in S:a_{\mathrm{iv}}=1\wedge \theta_{\mathrm{iv}}\leq \tau \right]}{\sum_{i\in P_{l}}w_{i}}$$

For reporting, all coverage rates are multiplied by 100 and expressed as percentages. The geometric coverage rate of the must-inspect surfaces is denoted by *C*_must_, while the effective coverage rates at 25° and 35° are denoted by *C*_25_ and *C*_35_, respectively. Hereafter, *C_m_*(*S*)≡*C*_must_(*S*). The notation *C_o_*_,*l*_(*S*) denotes the geometric coverage rate of the *l*-th optional-surface class. A tilde denotes the percentage form of a coverage rate, as shown in Equation (5):

(5) $${\tilde{C}}_{l}\left(S\right)=100C_{l}\left(S\right)$$

Accordingly, the must-inspect stage prioritizes increasing the geometric coverage and low-incidence-angle effective coverage of the must-inspect surfaces. After the must-inspect-surface viewpoint set has been fixed, the optional-surface stage supplements the coverage of each optional-surface class. Without changing the final viewpoint set, the visit-sequencing stage optimizes the path length subject to the keep-out-zone edge-feasibility constraint.

### 2.3. Overall Workflow

The proposed method comprises the six stages shown in Figure 1. The first three stages process the original STL model to generate target points with assigned task priorities and a finalized candidate pool. The viewpoint set for must-inspect surfaces and the supplementary set for optional surfaces are then determined sequentially. Finally, the visit sequence is optimized without changing the viewpoint set, yielding a complete acquisition plan.

## 3. Materials and Methods

### 3.1. Task-Oriented Inspection-Surface Prioritization

**Figure 2:**
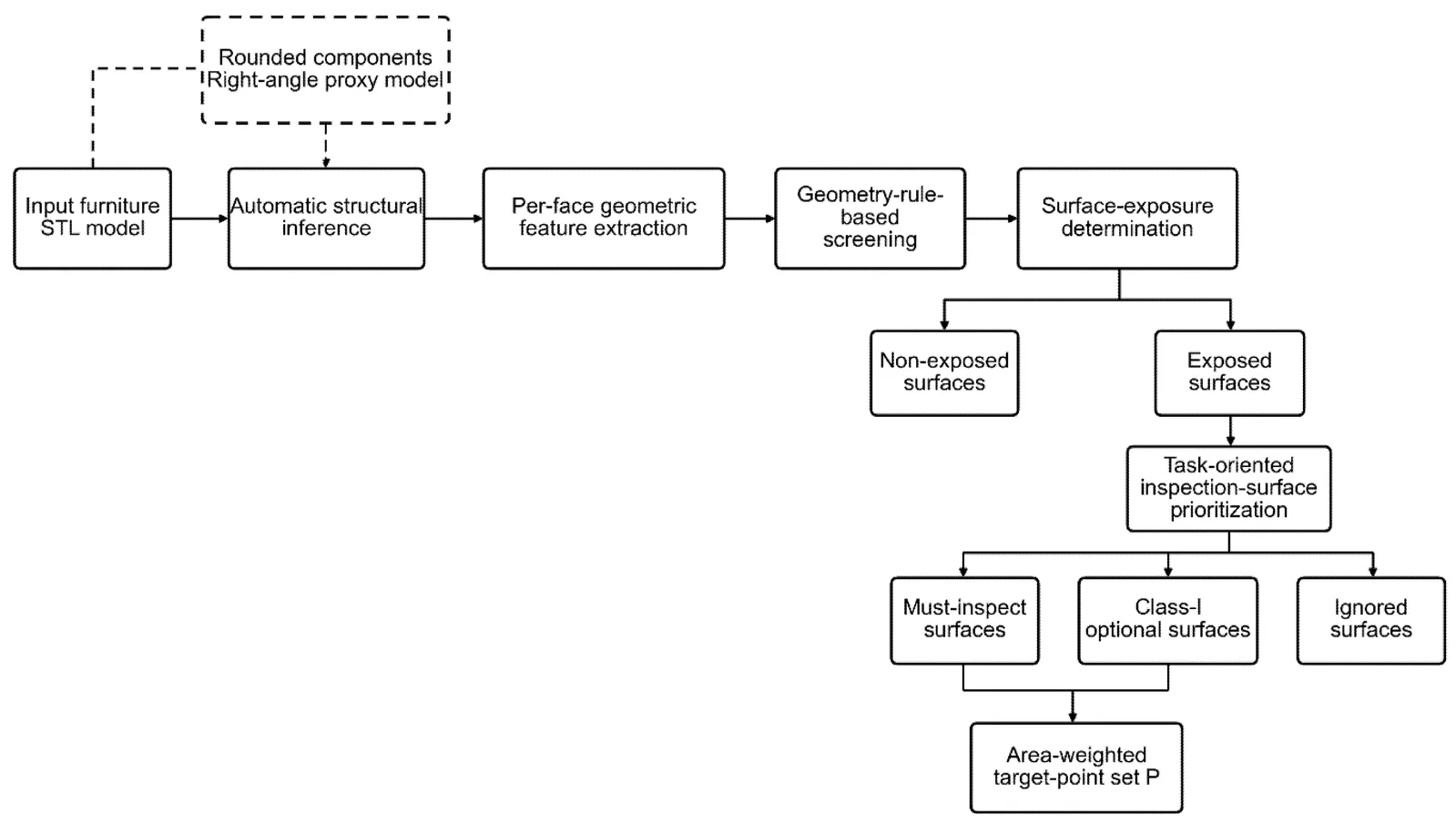
Geometry-rule-based inspection-surface prioritization workflow.

The combined process of determining surface exposure, assigning task labels, mapping the labels, and generating area-weighted target points is referred to as task-oriented inspection-surface prioritization. Based on the known STL geometry and inspection-task rules, this process classifies externally exposed surfaces as must-inspect, optional, or ignored surfaces, without relying on data-driven semantic segmentation. The algorithm infers the vertical direction, principal panel surfaces, and support hierarchy from the area distribution of horizontal surfaces. Non-exposed surfaces are then identified using face normals, relative heights, main-body projections, local continuous spans, and mesh-ray tests. The overall geometry-rule-based inspection-surface prioritization workflow is summarized in Figure 2.

**Figure 3:**
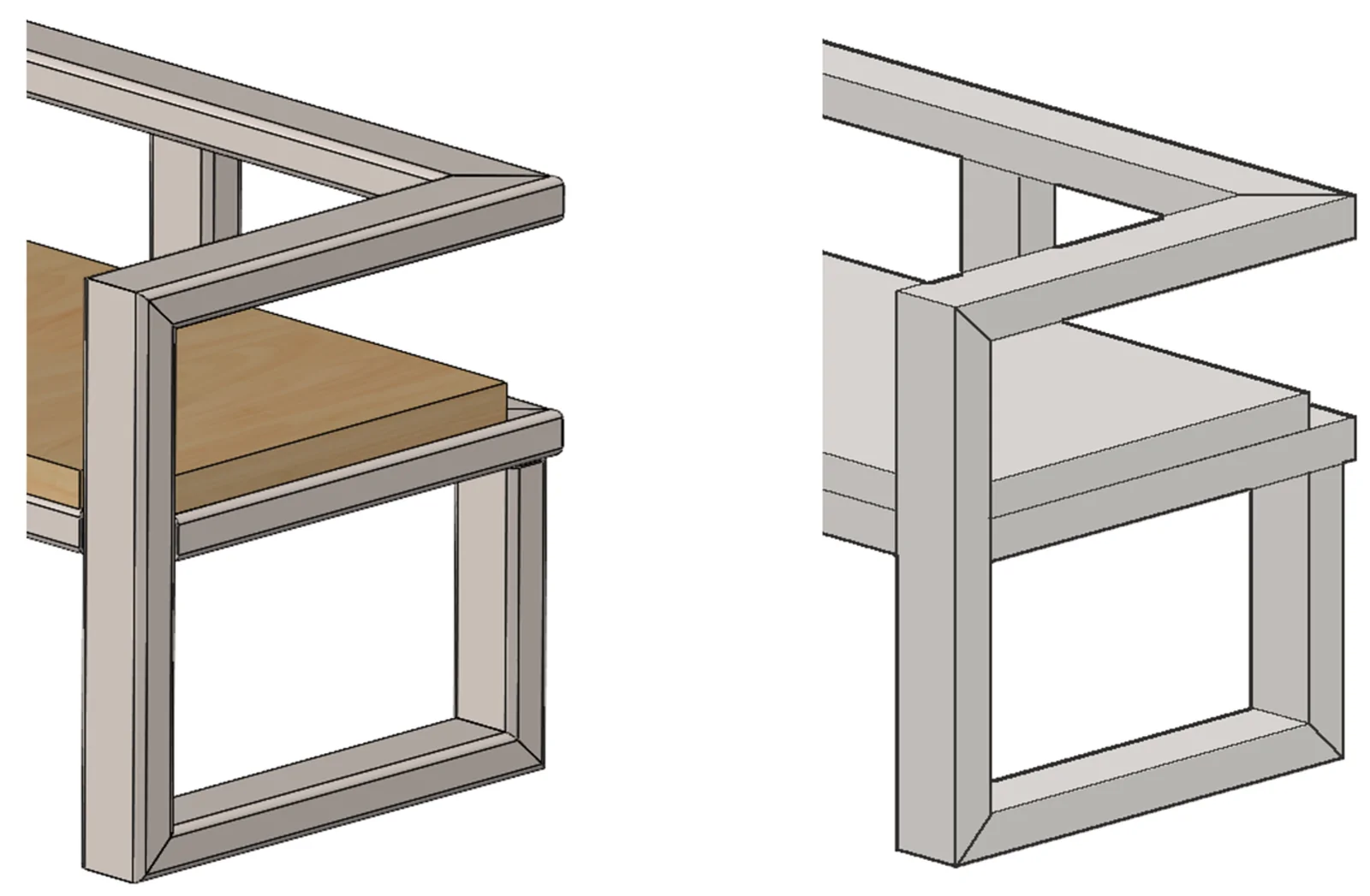
Bench with rounded corners and its right-angle proxy model.

For components with rounded corners, adjacent principal planes are first extended to construct a right-angle proxy model, as illustrated in Figure 3. After surface classification, the resulting labels are mapped back to the original mesh. The proxy model is used only for surface classification; target-point sampling and visibility evaluation are still performed on the original STL model.

After this process, the original furniture mesh is converted into a hierarchical inspection-surface target model comprising must-inspect, optional, and ignored surfaces. An area-weighted target-point set *P* is then generated on the surfaces included in the inspection task. This prioritization prevents low-priority regions from prematurely consuming the viewpoint budget allocated to must-inspect surfaces. Subsequent candidate-viewpoint generation, coverage evaluation, and observation-quality calculation are all performed using this target-point set.

### 3.2. Candidate-Pool Generation and Residual-Driven Iteration

Inspection-surface prioritization identifies the target regions to be observed but does not provide camera poses that satisfy the observation requirements. Therefore, an initial set of candidates with redundancy in both position and orientation is first generated. A residual-driven mechanism is then used to supplement the candidate pool with effective poses for boundary and occlusion-sensitive regions.

The initial candidates comprise target-anchor candidates and global-orbit candidates. The target points are aggregated using voxels to form structural anchors. Around each anchor, multi-level positional offsets and angular deflections are introduced along the surface-normal and local tangential directions, thereby generating an initial candidate pool that covers different positions and viewing directions. The specific ranges are provided in Section 4.2. Whether a candidate provides a valid observation is still determined by the actual distance between the target point and the camera center and by the valid observation-distance range of the corresponding model. After candidates with duplicate poses, insufficient heights, or locations within keep-out zones have been filtered out, the remaining candidates are further selected according to coarse coverage, observation quality, and spatial diversity.

A candidate pool generated in a single pass tends to contain many near-duplicate poses in open regions while undersampling boundary and occlusion-sensitive regions. Therefore, the candidate pool is iteratively refined through an “evaluation–diagnostic selection–residual supplementation” process. For the must-inspect surfaces, uncovered target points, target points covered by only one viewpoint, and target points whose best incidence angle exceeds 25° are treated as residuals. For each optional-surface class, uncovered points are extracted separately and clustered to generate local candidates.

For a visible target point, the basic projection quality *q_iv_* combines three normalized components associated with the incidence angle, position within the field of view, and observation distance, as defined by Equations (6)–(9):

(6) $$q_{\mathrm{iv}}=0.52q_{\mathrm{iv}}^{\theta }+0.28q_{\mathrm{iv}}^{f}+0.20q_{\mathrm{iv}}^{d}$$

(7) qivθ=clip θmax−θivθmax,0,1,  θmax=65∘

(8) qivf=clip 1−max ξivtanαh/2,ηivtanαv/2,0,1

(9) qivd=1−clip div−d0dmax−dmin/2,0,1,  d0=dmin+dmax2

where

clip(x,0,1) = min(max(*x*,0),1)

Here, *θ_iv_* is the incidence angle of target point *i* at candidate viewpoint *v*, and *θ*_max_ = 65^∘^. The variables *ξ_iv_* and *η_iv_* are the horizontal and vertical coordinates, respectively, of the projected target point after normalization by the camera optical-axis depth $z_{\mathrm{iv}}^{c}$. The variables *α_h_* and *α_v_* are the horizontal and vertical camera field-of-view angles, respectively. The Euclidean distance from the camera center to the target point is given by Equation (10):

(10) $$d_{\mathrm{iv}}={∥x_{i}-c_{v}∥}_{2}.$$

The variables *d*_min_ and *d*_max_ are the lower and upper bounds, respectively, of the valid observation-distance range for target points of the corresponding model, and *d*_0_ is their midpoint. All three components are normalized to [0, 1] and respectively quantify incidence-angle quality, proximity to the center of the field of view, and observation-distance quality. A higher value indicates more favorable imaging conditions. For an invisible target point, *q_iv_* = 0. The weights 0.52, 0.28, and 0.20 in Equation (6) place the greatest emphasis on incidence-angle quality while also accounting for FOV-center and observation-distance quality.

The residual-driven candidate-pool iteration is presented in Algorithm 1.
**Algorithm 1.** Residual-driven candidate-pool iteration1:**Input:** Hierarchical inspection-surface target-point set *P*, number of optional-surface classes *L*, maximum number of residual-supplementation rounds *R*_max_, and per-round candidate-pool retention limit *N*_keep_.2:**Output:** Final candidate pool V^*^.3:*V^(0)^* ← **Filter**(**GenerateInitial**(*P*))4:*A^(0)^* ← **MeshRay**(*V^(0)^*, *P*)5:*S*_diag_^(0)^ ← **DiagnosticSelect**(*V*^(0)^, *A*^(0)^)6:**for** *r* = 0, …, *R_max_*−1 **do**7: *R*_m_^(r)^ ← **ResidualMain**(*S*_diag_^(r)^, *A*^(r)^)8: *R*_o,l_^(r)^ ← **ResidualOptional**(*S*_diag_^(r)^, *A*^(r)^, *l*), *l* = 1, …, *L*9: *B^(r)^* ← **ClusterByLabel**(*R_m_^(r)^*, *R_o,1_^(r)^*, …, *R_o,L_^(r)^*)10: Δ*V^(r)^* ← **Filter**(**GenerateLocal**(*B^(r)^*))11: Δ*A^(r)^* ← **MeshRay**(Δ*V^(r)^*, *P*)12: (*V*^(r+1)^, *A*^(r+1)^) ← **Update**(       *V*^(r)^, *A*^(r)^, Δ*V*^(r)^, Δ*A*^(r)^, *S*_diag_^(r)^, *N*_keep_)13: *S*_diag_^(r+1)^ ← **DiagnosticSelect**(*V*^(r+1)^, *A*^(r+1)^)14:**end for**15:V^*^ ← *V(R_max_)*16:**return** V^*^

The temporary set $S_{\mathrm{diag}}^{\left(r\right)}$ is used only to identify uncovered, single-view-covered, and low-quality-observation residuals in the current candidate pool. DiagnosticSelect progressively selects candidate viewpoints according to their area-weighted residual reduction. Here, $\Delta R\left(v|S_{\mathrm{diag}}^{\left(r\right)}\right)$ denotes the reduction in diagnostic residuals obtained by adding candidate viewpoint *v*. The three residual types—uncovered, single-view-covered, and low-quality-observation residuals—are all weighted by the representative areas of the target points and jointly constitute the total diagnostic residual. Accordingly, Δ*R* is the reduction in this total before and after the candidate viewpoint is added. The selection process stops when none of the remaining candidates can further reduce these residuals. Therefore, the size of the diagnostic set may vary across iterations and does not represent the final number of must-inspect-surface viewpoints. Algorithm 1 ultimately outputs the final candidate pool *V*^⋆^, whereas the number of must-inspect-surface viewpoints *K_m_* is determined by the stage-branch comparison described in Section 3.3. Diagnostic candidate selection is defined by Equation (11).

(11) $$v^{⋆}=\mathrm{arg max}\Delta R(v|S_{\mathrm{diag}}^{\left(r\right)})\;v\in V^{\left(r\right)}\setminus S_{\mathrm{diag}}^{\left(r\right)}$$

The selection process stops when the maximum residual reduction is zero. *A*^(*r*)^ records the coverage, incidence angles, and projection quality of the active pool in iteration *r*. Update merges the existing and newly generated candidates, prioritizing the diagnostic set, candidates that contribute to scarce residuals, and candidates with high combined coverage and observation quality. The active pool is then limited to *N*_keep_ candidates. The specific retention limit and maximum number of supplementation rounds are provided in Section 4.2. After the iteration is complete, the final candidate pool *V*^⋆^ is obtained and used as the common candidate space for subsequent viewpoint selection.

### 3.3. Viewpoint Selection and Replacement for Must-Inspect Surfaces

#### 3.3.1. Quality-Aware Incremental Viewpoint Selection

The final candidate pool provides a relatively complete set of candidate viewing directions but still contains a large number of redundant poses. A limited number of viewpoints must therefore be selected to form the viewpoint set for must-inspect surfaces. The must-inspect stage seeks not only to increase geometric coverage but also to prioritize lower incidence angles and better projection conditions. Accordingly, viewpoints are selected incrementally using a quality-aware marginal score. The score simultaneously considers geometric coverage gain, 25° and 35° effective coverage gains, improvements in the best incidence angle and projection quality, and high-incidence-angle, low-quality coverage gain.

For a target point *i* on the must-inspect surfaces, its best incidence angle under viewpoint set *S*, denoted by $\theta_{i}^{⋆}\left(S\right)$, is the minimum *θ_iv_* among all visible observations, and its best projection quality $q_{i}^{⋆}\left(S\right)$ is the maximum *q_iv_*. For an uncovered target point, these quantities are set to *θ*_max_ and 0, respectively. All related area-weighted calculations are performed over the must-inspect target-point set *P_m_*. The area-weighted improvements provided by candidate viewpoint *v* are defined by Equations (12) and (13):

(12) $$\Delta \Theta \left(v|S\right)=\frac{\sum_{i}w_{i}a_{\mathrm{iv}}\max\left[\theta_{i}^{⋆}\left(S\right)-\theta_{\mathrm{iv}},0\right]}{\sum_{i}w_{i}}$$

(13) $$\Delta Q\left(v|S\right)=100\;\frac{\sum_{i}w_{i}\max\left[q_{\mathrm{iv}}-q_{i}^{⋆}\left(S\right),0\right]}{\sum_{i}w_{i}}$$

Here, Δ*Θ* is measured in degrees, whereas Δ*Q* and the coverage gains are measured in percentage points. The high-incidence-angle, low-quality coverage gain is defined as the nonnegative difference between the geometric coverage gain and the 35° effective coverage gain. The overall candidate score is defined by Equation (14):

*s*(*v*∣*S*) = 8Δ*C*_25_ + 3Δ*C*_35_ + 0.45Δ*C* + 0.20Δ*Θ* + 1.5Δ*Q* − 5.5Δ*C*_bad_ (14)

While retaining geometric coverage as the primary driving factor, this score favors gains in low-incidence-angle effective coverage and suppresses candidates whose scores rely primarily on high-incidence-angle coverage. At each step, the candidate with the highest score among those providing a positive geometric coverage gain is selected. The same weights are used for all four full-planning models.

This process can rapidly establish the basic coverage of the must-inspect surfaces. However, for the small number of uncovered and low-quality residuals remaining in fragmented boundary regions, simply continuing to add viewpoints may not be the most effective solution.

#### 3.3.2. Residual-Driven Local Pose Replacement

Local pose replacement is used to address the small number of uncovered residual regions left by the must-inspect stage. It adjusts some existing poses while keeping the current number of viewpoints unchanged. The method determines the viewpoints to be replaced based on the residual distribution and the coverage contributions of the existing viewpoints, and generates local pose variants around the aggregated residual regions. These local variants include perturbations of the viewpoint position, adjustments to the look-at direction, and a translation of the entire pose toward the residual region. The subscript *m* denotes the must-inspect target category.

The set-level quality function used for replacement is defined by Equation (15):

(15) $$\Psi_{m}\left(S\right)=10{\tilde{C}}_{m}\left(S\right)+4{\tilde{C}}_{m,35}\left(S\right)+{\tilde{C}}_{m,25}\left(S\right)-0.20{\bar{\theta }}_{m}\left(S\right)-0.10\theta_{m,95}\left(S\right)$$

Here, ${\bar{\theta }}_{m}\left(S\right)\;$ and *θ_m_*_,95_(*S*) denote the area-weighted mean and 95th-percentile best incidence angles of the must-inspect target points under viewpoint set *S*, respectively. The five coefficients in Equation (15) balance geometric coverage, low-incidence-angle effective coverage, and incidence-angle statistics.

Let $S_{m}^{\left(r-1\right)}$ denote the current must-inspect-surface viewpoint set before the *r*-th replacement, and let $T_{m}^{\left(r\right)}$ denote the trial set obtained by replacing one original viewpoint with a local candidate. Only one viewpoint is considered for replacement at each attempt. A replacement is accepted only when the overall quality improves, the decrease in must-inspect coverage does not exceed *ε_C_*, and the increase in the 95th-percentile incidence angle does not exceed *ε_θ_*. The specific conditions are given by Equations (16)–(18):

(16) $$\Psi_{m}\;\left(T_{m}^{\left(r\right)}\right)>\Psi_{m}\;\left(S_{m}^{\left(r-1\right)}\right)$$

(17) $${\tilde{C}}_{m}\left(T_{m}^{\left(r\right)}\right)\geq {\tilde{C}}_{m}\left(S_{m}^{\left(r-1\right)}\right)-\varepsilon_{C}$$

(18) $$\theta_{m,95}\left(T_{m}^{\left(r\right)}\right)\leq \theta_{m,95}\left(S_{m}^{\left(r-1\right)}\right)+\varepsilon_{\theta }$$

The parameters ε_C_ and ε_θ_ limit the degradation in must-inspect coverage and the 95th-percentile incidence angle, respectively. Their specific values are given in Section 4.2. Each original viewpoint can be replaced at most once, and the replacement process keeps the size of the current viewpoint set unchanged.

For a current set containing k viewpoints, a local-replacement branch $S_{\mathrm{ref}}^{k}$, which retains the same number of viewpoints, and a continued viewpoint-addition branch $S_{\mathrm{add}}^{k+1}$, which increases the number of viewpoints to *k* + 1, are constructed. If Equation (19) holds,

(19) $$C_{m}\left(S_{\mathrm{ref}}^{k}\right)\geq C_{m}\left(S_{\mathrm{add}}^{k+1}\right)$$

the local-replacement result containing fewer viewpoints is retained, the expansion of the must-inspect-surface viewpoint set is stopped, and *K_m_* is set to *k*. Otherwise, another viewpoint is added and the comparison is repeated. The final must-inspect-surface viewpoint set $S_{m}^{⋆}$ is thus obtained. Thus, *K_m_* is the viewpoint count at which the local-replacement branch is retained over continued viewpoint addition under the stage-comparison condition.

$$|S_{m}^{⋆}|=K_{m}.$$

### 3.4. Supplementary Viewpoint Selection for Optional Surfaces

Once the must-inspect-surface viewpoint set has been determined, the planning focus shifts from priority coverage of the must-inspect surfaces to supplementary acquisition of the optional-surface regions. Because the current coverage and remaining coverage potential differ among optional-surface categories, ranking candidates solely by total gain may cause newly added viewpoints to concentrate on categories that have larger areas or are easier to cover. Therefore, candidates that provide a positive gain for at least one optional-surface category are selected from the final candidate pool, while both the total coverage gain across categories and the minimum category gain are considered.

For the *l*-th optional-surface category, the additional coverage *g_l_*(*v*∣*S*) provided by candidate *v* and the urgency weight *u_l_*(*S*), which increases as the current coverage decreases, are defined by Equation (20):

(20) $$u_{l}\left(S\right)=1+\frac{100-{\tilde{C}}_{o,l}\left(S\right)}{100},\;\;g_{l}\left(v|S\right)={\tilde{C}}_{o,l}\left(S\cup \{v\}\right)-{\tilde{C}}_{o,l}\left(S\right)$$

The joint score combines the weighted total gain across all categories and the minimum gain among categories. With *β* = 0.35, the joint score is defined by Equation (21):

(21) $$G\left(v|S\right)=\sum_{l=1}^{L}u_{l}\left(S\right)g_{l}\left(v|S\right)+\beta \underset{1\leq l\leq L}{\min}g_{l}\left(v|S\right)$$

At each step, the candidate with the highest joint score and a positive gain is selected. The minimum-gain term is used to improve coverage balance among the different optional-surface categories. The balance coefficient β controls the contribution of the minimum-category gain relative to the total gain; β = 0.35 is used as the baseline setting.

The score *G*(*v*∣*S*) is used only for candidate ranking. The L-method [33] uses the unweighted aggregate coverage gain produced by each selected candidate, as defined in Equation (22):

(22) $$H_{j}=\sum_{l=1}^{L}g_{l}\left({\hat{v}}_{j}|S^{\left(j-1\right)}\right)$$

Here, ${\hat{v}}_{j}$ is the *j*-th selected viewpoint in the supplementary-viewpoint sequence, and *S*^(*j*−1)^ is the current viewpoint set before ${\hat{v}}_{j}\;$ is added. Supplementary viewpoints continue to be selected until no candidate provides a positive gain, yielding the complete gain sequence $H_{1},\ldots ,H_{N_{o}}$. The supplementary-viewpoint indices and cumulative actual gains are then normalized. Linear least-squares fitting is performed on both sides of each feasible breakpoint, and the breakpoint with the minimum sample-size-weighted root-mean-square error is selected as the knee point *k*^⋆^. The first *k*^⋆^ viewpoints in the supplementary-viewpoint sequence are ultimately retained.

This process yields the optional-surface supplementary set *S_o_*, and the final viewpoint set is given by Equation (23):

(23) $$S=S_{m}^{⋆}\cup S_{o}.$$

At this point, the viewpoint positions and their number have been determined, and only the visit sequence is optimized in the subsequent stage.

### 3.5. Keep-Out-Zone-Constrained Visit Sequencing

After the final viewpoint set has been determined, the visit-sequencing stage optimizes only its open-path visit sequence, without changing the viewpoint positions or their number. A complete graph is constructed with the camera centers as nodes. For each pair of viewpoints, the Euclidean distance and the intersection status of their straight-line connection with the keep-out zones are calculated. The keep-out zones are constructed by expanding the axis-aligned bounding box of the furniture model outward by 25 mm in each coordinate direction. The 25 mm expansion used in this study can be adjusted according to furniture size, imaging hardware, and the required safety margin.

Let *d_ij_* denote the Euclidean distance between the camera centers of viewpoints *v_i_* and *v_j_*, and let *b_ij_* denote the keep-out-zone intersection indicator. If the line segment connecting the two camera centers intersects the interior of any predefined keep-out zone, then *b_ij_* = 1; otherwise, *b_ij_* = 0. The feasible edge set is defined by Equation (24):

*E_feas_* = {(*i*,*j*) ∣ *b_ij_* = 0} (24)

Edges with *b_ij_* = 1 are excluded from the search graph and are unavailable during both ACO construction and 2-opt local optimization. The feasible edge set *E_feas_* therefore contains only viewpoint connections that do not intersect the predefined keep-out zones.

Open-path ACO [34] is used to search for the visit sequence. During ACO construction, transition probabilities are normalized only over unvisited feasible neighbors. In 2-opt refinement, a candidate exchange is permitted only if all newly introduced edges belong to *E_feas_*.

The final path length is calculated using the Euclidean distance, while the number of keep-out-zone-intersecting edges in the visit sequence is also recorded.

## 4. Experimental Setup

### 4.1. Experimental Models and Inspection-Surface Prioritization

**Figure 4:**
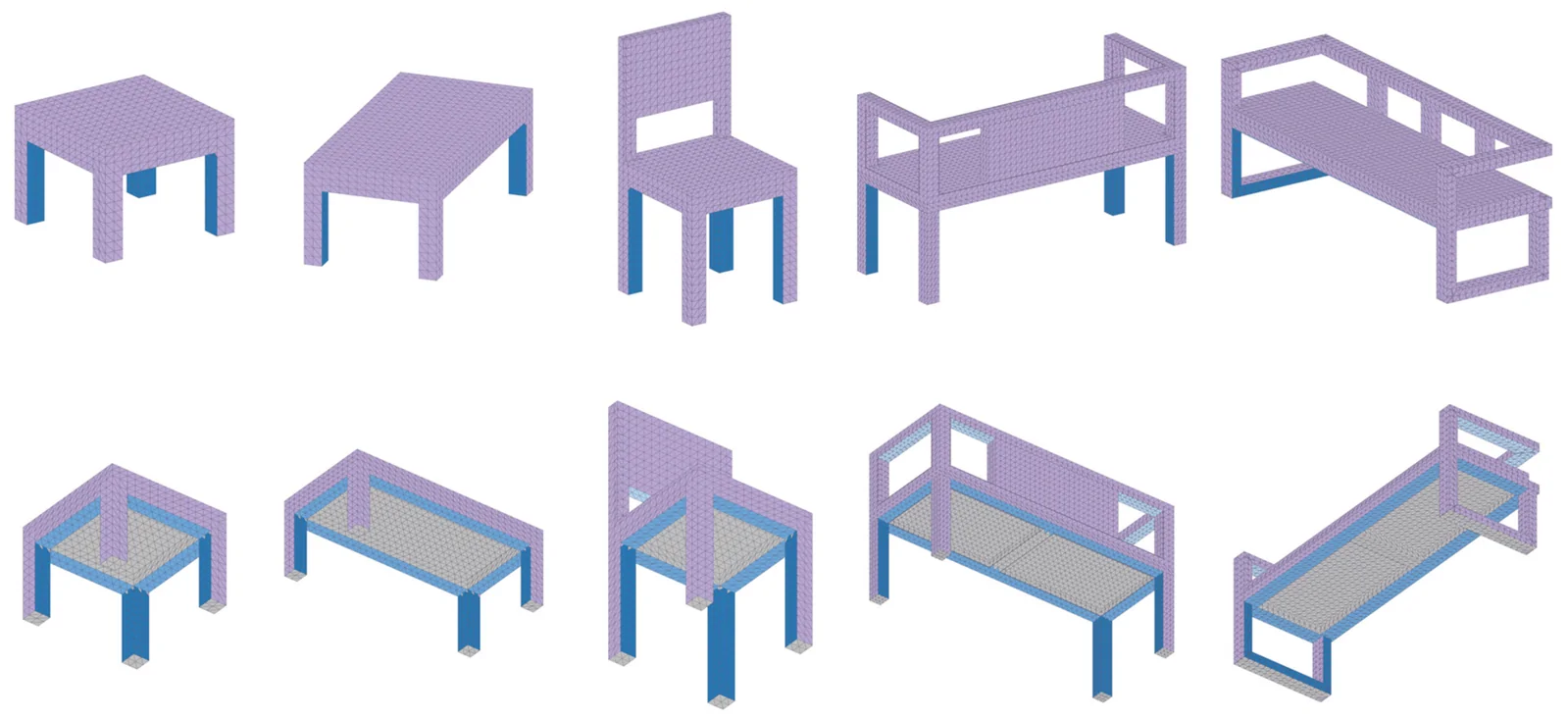
Inspection-surface prioritization for five furniture models.

The experiments applied a unified inspection-surface prioritization procedure to five STL models, a regular table, a long table, a chair, and two bench structures, as shown in Figure 4. All five models were processed using the same rules for structural inference, geometric screening, and surface-exposure determination, without manual adjustment of the inspection-surface prioritization rules for any individual model. Of these, four models—the regular table, long table, chair, and the bench—were further used in the full-planning, comparative, ablation, and path experiments. For models with rounded corners, inspection-surface prioritization was first performed using a right-angle proxy model, after which the labels were mapped back to the original mesh.

### 4.2. Experimental Parameters and Evaluation Metrics

Area-weighted target points were generated with a spacing of 3 mm on both the must-inspect and optional surfaces. The horizontal and vertical camera field-of-view angles were 65° and 50°, respectively, and the maximum incidence angle was 65°. The valid observation-distance range for target points was 50–300 mm for the regular table and long table, 50–350 mm for the chair, and 50–500 mm for the bench. Visibility was consistently evaluated using one-sided triangle-mesh ray casting, while numerical self-intersections within the neighborhoods of the camera and target-point endpoints were ignored. The candidate-generation offset ranges for the must-inspect and optional surfaces were 80–340 mm and 75–330 mm, respectively. The number of iterative supplementation rounds was determined based on gain saturation; *R*_max_ was set to two, three, three, and four for the regular table, long table, chair, and bench, respectively. The active candidate-pool retention limit *N*_keep_ was set to 1100 for all four models.

The geometric coverage, 25° effective coverage, and 35° effective coverage of the must-inspect surfaces are denoted by *C*_must_, *C*_25_, and *C*_35_, respectively. Smaller values of the area-weighted mean and 95th percentile of the best incidence angles indicate that the viewing directions are closer to the surface normals. The local-replacement protection bounds were set to *ε_C_* = 0.02 percentage points and *ε_θ_* = 0.75°. Here, *ε_C_* = 0.02 percentage points and *ε_θ_* = 0.75° limit the allowable degradation in geometric coverage and the high-percentile incidence angle during replacement. The sensitivity of the main weighting coefficients and protection thresholds is analyzed in Section 5.6.

Path performance was evaluated using the open-path length and the number of keep-out-zone-intersecting edges. Edges whose straight-line camera-center connections intersected the expanded keep-out AABB were treated as infeasible during both ACO construction and 2-opt refinement. ACO was run for 80 iterations, with the number of ants set to the larger of the viewpoint count and 24. The randomized experiments used 20 sets of paired seeds. Before optimization, the feasible graph was checked for open-path feasibility, and a complete feasible visit sequence existed for all four full-planning models.

The experiments were conducted in a Python 3.11.9 environment using NumPy 2.4.4, pandas 3.0.2, SciPy 1.17.1, trimesh 4.12.2, and Matplotlib 3.10.9.

### 4.3. Baseline Methods and Statistical Protocol

The proposed method was evaluated from four aspects: must-inspect-surface viewpoint selection, the effects of key components, supplementary viewpoint selection for optional surfaces, and visit sequencing.

Under the same frozen candidate pool and viewpoint budget, two deterministic baselines were used. GSC maximizes the incremental must-inspect coverage at each step. Q-NBV adds projection-quality gain and a redundant-coverage penalty to the incremental coverage. Neither baseline performs local pose replacement. All selectors use *K_m_* viewpoints, where *K_m_* is determined by the stage-branch comparison of the proposed method. The Q-NBV score is defined by Equation (25):

*s*_Q−NBV_(*v*∣*S*)=Δ*C*(*v*∣*S*) + 0.35Δ*Q*(*v*∣*S*) − 0.10Δ*C*_rep_(*v*∣*S*) (25)

All three terms are normalized by the total area of the must-inspect surfaces. This fixed-budget comparison isolates the selection quality of the different viewpoint-selection strategies rather than their ability to determine the number of viewpoints independently.

GA and BPSO use different strategies to search for viewpoint subsets. They share the frozen candidate pool, must-inspect target points, and viewpoint count *K_m_* with the proposed method. Each GA individual or BPSO particle represents a complete subset *S* containing *K_m_* viewpoints. The fitness of each subset is calculated from its area-weighted coverage, incidence-angle gain, and projection quality over the must-inspect target points, rather than from the marginal gains used in sequential viewpoint selection. GA uses a fixed-length index representation, whereas BPSO uses Gumbel top-*K_m_* sampling to retain exactly *K_m_* viewpoints. Both methods use 50 individuals or particles and run for a maximum of 100 generations, with early termination if no improvement occurs for 25 consecutive generations. Both methods use the set-level fitness function defined by Equation (26) and are independently run 20 times:

*F*(*S*) = 8*C*_25_(*S*) + 3*C*_35_(*S*) + 0.45*C_must_*(*S*) + 0.20*G_θ_*(*S*) + 1.5*Q*(*S*) − 5.5*C_bad_*(*S*) (26)

*C_bad_*(*S*) = *max*[*C_must_*(*S*) − *C*_35_(*S*),0] (27)

Here, *C*_must_(*S*), *C*_25_(*S*), and *C*_35_(*S*) denote the geometric coverage, 25° effective coverage, and 35° effective coverage, respectively, of viewpoint set *S* over the must-inspect target points. *G_θ_*(*S*) and *Q*(*S*) denote the set-level best-incidence-angle gain and projection quality, respectively. Specifically, *G_θ_*(*S*) is the area-weighted mean of $\theta_{\max}-\theta_{i}^{⋆}\left(S\right)$, and *Q*(*S*) is 100 times the area-weighted mean of $q_{i}^{⋆}\left(S\right)$. In Equation (27), *C*_bad_(*S*) penalizes low-quality geometric coverage with incidence angles greater than 35°. The numerical scales of all terms are consistent with those used in Section 3.3. The weights are held constant across all four full-planning models and all independent runs.

The must-inspect selector comparisons use frozen candidate pools obtained through the unified candidate-generation procedure. The pool sizes are 1202, 2415, 1370, and 897 for the regular table, long table, chair, and bench, respectively. These frozen pools are used only for the unified comparison of GSC, Q-NBV, the proposed selector, and GA/BPSO. They differ from the active candidate pools constrained during the iterative stage and do not include new poses generated by subsequent local pose replacement.

Local pose replacement generates alternative poses by perturbing the camera position and look-at direction. In the component-ablation experiments, candidate-pool iteration, local pose replacement, and observation-quality control are removed individually under a fixed total viewpoint count, while the remainder of the workflow is kept unchanged. The optional-surface experiments fix the must-inspect viewpoint set and the final candidate pool, generate the complete positive-gain sequence, and determine the knee point using the weighted two-segment L-method.

The path experiments compare the Euclidean-distance ACO+2-opt with keep-out-zone-constrained ACO+2-opt. The two methods use the same viewpoint sets, the same set of 20 paired random seeds, the same ACO parameters, and the same 2-opt procedure, thereby forming strictly paired experiments. For each of the four models, two-sided Wilcoxon signed-rank tests are performed separately on the path length and the number of keep-out-zone-intersecting edges, resulting in eight tests in total. The Holm correction is applied across all eight tests, with the significance level set to 0.05.

## 5. Results and Discussion

This section first presents the final coverage, observation quality, and optional-surface supplementation results of the complete method for four furniture models. It then presents the incremental selection process for must-inspect-surface viewpoints, compares the fixed-count selection results, and evaluates the effects of candidate-pool iteration, observation-quality control, and local pose replacement through component-ablation experiments. The relationship between optional-surface coverage gain and the number of supplementary viewpoints is subsequently examined. Finally, the path length and the number of keep-out-zone-intersecting edges obtained using Euclidean-distance and keep-out-zone-constrained sequencing are compared. A parameter sensitivity analysis is then conducted on the long-table model, followed by a viewpoint-budget sensitivity analysis for all four models.

### 5.1. Overall Planning Results

Table 1 presents the final planning results for the four furniture models. The must-inspect-surface viewpoint sets for the regular table, long table, chair, and bench contained 14, 15, 22, and 55 viewpoints, respectively. After supplementary viewpoints for optional surfaces were added, the final viewpoint sets contained 19, 23, 30, and 72 viewpoints. The geometric coverage rates of the must-inspect surfaces were 100.00%, 100.00%, 99.86%, and 98.16%, respectively. At these viewpoint counts, the 35° effective coverage rates ranged from 97.80% to 99.86%, indicating that most must-inspect regions were observed at incidence angles no greater than 35°.

The supplementary-viewpoint stage additionally covered optional regions not included in the must-inspect task. The mean coverage rates across the optional-surface classes of the regular table, long table, chair, and bench were 87.50%, 88.14%, 90.11%, and 91.57%, respectively. These results indicate that the joint score balanced the coverage gains of different optional-surface classes under a limited number of supplementary viewpoints.

**Figure 5:**
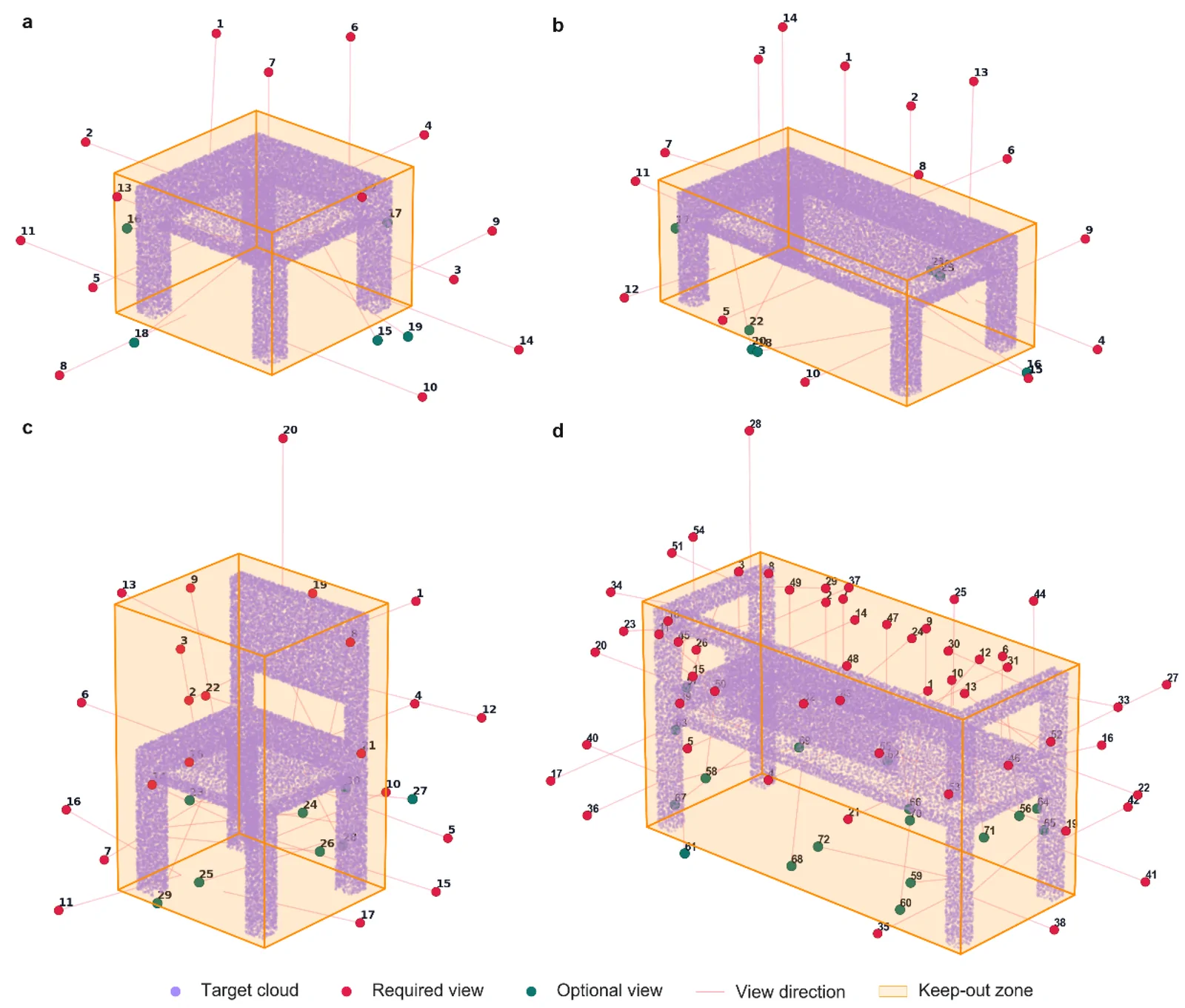
Final viewpoint distributions for four furniture models: (**a**–**d**) regular table, long table, chair, and bench, respectively.

Figure 5 shows the spatial distributions of the final viewpoints. The must-inspect-surface viewpoints are located mainly around and above the furniture and are used to acquire larger appearance surfaces. The optional-surface viewpoints are concentrated mainly in less directly visible regions. Their distributions reflect the inspection-surface priorities and local occlusion structures of the respective models.

### 5.2. Must-Inspect-Surface Viewpoint Selection at Fixed Viewpoint Counts

This section examines whether quality-aware scoring can accumulate low-incidence-angle effective coverage more rapidly while maintaining geometric coverage when the number of must-inspect-surface viewpoints is fixed at *K_m_*. For all four models, *K_m_* was determined by comparing the local pose replacement branch with the continued viewpoint addition branch. For each model, all methods used the same frozen candidate pool, must-inspect target points, and visibility matrix. None of the methods included the subsequent local pose replacement or optional-surface supplementation stages. The deterministic baselines were GSC and Q-NBV, whereas the stochastic baselines were GA and BPSO, each independently run 20 times using random seeds 0–19.

**Figure 6:**
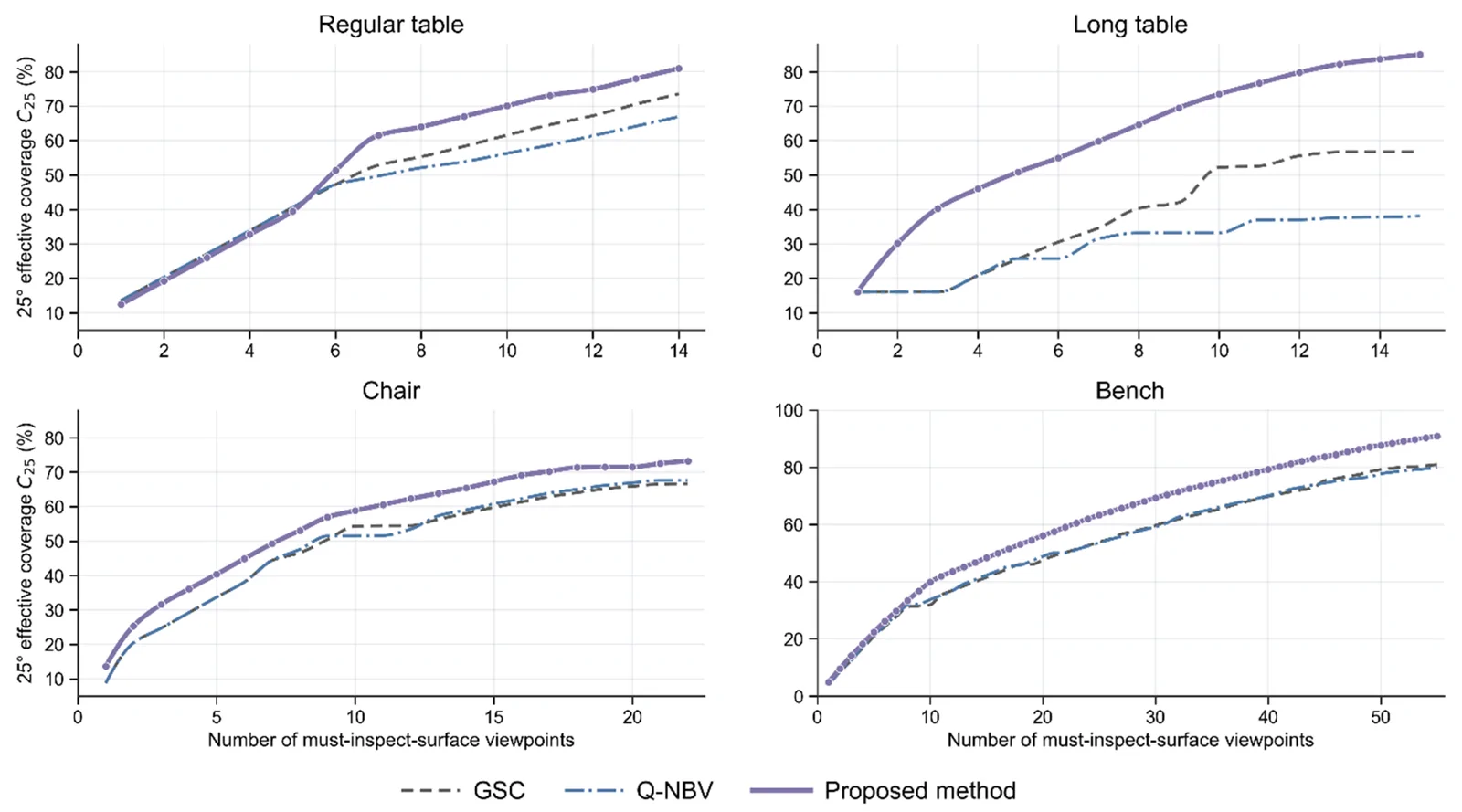
Effective coverage at 25° versus must-inspect-surface viewpoint count.

Figure 6 shows that the proposed method achieved higher values for most viewpoint counts for all four models, with the largest difference observed for the long table. As the viewpoint count increased, the curve of the proposed method generally rose more rapidly. This result indicates that, at the same viewpoint count, quality-aware scoring preferentially selects candidates that facilitate low-incidence-angle observation rather than simply pursuing the rapid accumulation of geometric coverage.

To further compare geometric coverage and angular quality at the fixed-count endpoints, Table 2 summarizes *C*_must_, *C*_25_, *C*_35_, the mean best incidence angle, and *P*_95_ (95th-percentile best incidence angle) for the three deterministic methods. In the comparative tables, the best values are shown in bold.

At the fixed-count endpoints, the must-inspect coverage values of the proposed method were 99.90%, 99.90%, 98.01%, and 96.31% for the regular table, long table, chair, and bench, respectively. The corresponding 25° effective coverage values were 80.96%, 84.99%, 73.24%, and 90.98%, exceeding those of the best deterministic baselines by 7.43, 28.30, 5.63, and 10.02 percentage points, respectively. The most pronounced improvement was observed for the long table. Compared with GSC, its 25° effective coverage increased from 56.69% to 84.99%, the mean best incidence angle decreased from 23.82° to 16.87°, and *P*_95_ decreased from 46.96° to 30.16°. For the chair, Q-NBV and GSC achieved slightly higher geometric coverage, whereas the proposed method achieved higher 25° effective coverage and lower incidence-angle metrics. For the bench, GSC achieved slightly higher geometric coverage, whereas the proposed method achieved higher 25° and 35° effective coverage and lower incidence-angle metrics.

Table 3 further compares the endpoint metrics of the proposed method, GA, and BPSO at matched must-inspect-surface viewpoint counts. For the proposed method, a single deterministic result is obtained using the fixed candidate pool and parameter settings. GA and BPSO were independently run 20 times using random seeds 0–19, and their means and sample standard deviations are reported.

**Figure 7:**
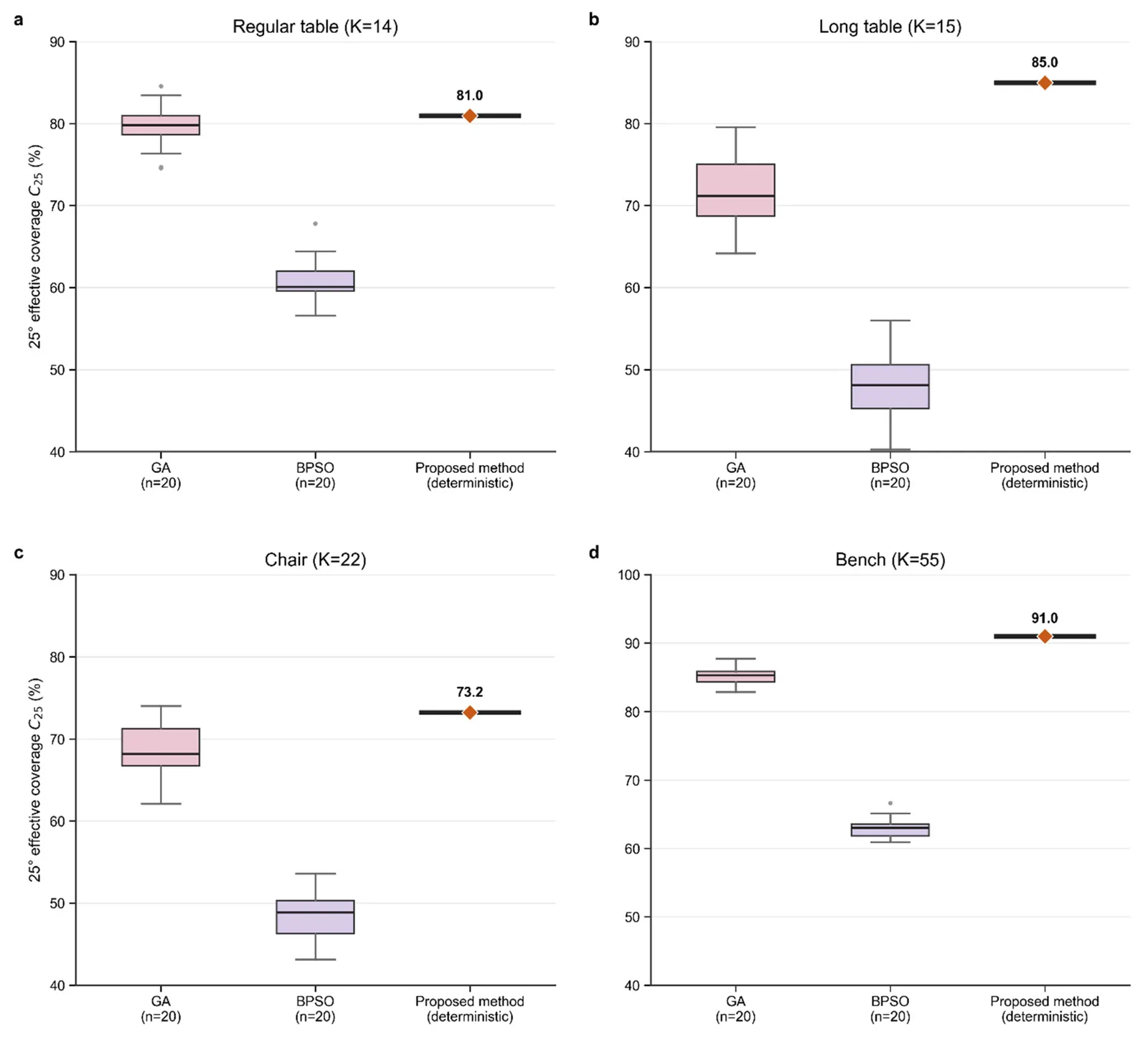
Comparison of 25° effective coverage among the proposed selector, GA, and BPSO.

The stochastic comparisons in Figure 7 are consistent with the incremental accumulation trends shown in Figure 6. Based on the raw results, the 25° effective coverage values of the proposed method exceeded the 20-run means of GA by 1.19, 13.16, 4.75, and 5.80 percentage points for the regular table, long table, chair, and bench, respectively, and exceeded the corresponding BPSO means by 20.09, 36.72, 24.78, and 27.92 percentage points. For the four models, the proposed value exceeded the corresponding values in 15, 20, 19, and 20 of the 20 GA runs, respectively, and exceeded those of all 20 BPSO runs for all four models.

Figure 6 and Figure 7 and Table 2 and Table 3 collectively show that the proposed method achieved higher geometric and effective coverage than GA and BPSO for all four models, while the mean best incidence angles were generally comparable. These results indicate that the principal advantage of the proposed method is that it enables more selected viewpoints to provide effective observations at low incidence angles while maintaining high geometric coverage. The geometric-coverage and angular-quality residuals remaining after the initial must-inspect-surface viewpoint selection are further reduced through residual-driven local pose replacement, as examined in Section 5.3.

### 5.3. Ablation Results for Key Components

Table 4 presents the ablation results for key components at fixed total viewpoint counts. For all four models, the total viewpoint counts are those listed in Table 1: 19, 23, 30, and 72 for the regular table, long table, chair, and bench, respectively. In each experiment, only one of the following components was removed: candidate-pool iteration, local pose replacement, or observation-quality control. All other procedures and parameters were kept unchanged. The differences shown in the table therefore mainly reflect the effects of the individual components on the final coverage profile.

When candidate-pool iteration was removed, the must-inspect coverage values of the regular table, long table, chair, and bench were 97.68%, 78.25%, 51.25%, and 60.02%, respectively, while the corresponding 35° effective coverage values were 97.12%, 64.15%, 36.87%, and 59.91%. The larger reductions observed for the long table, chair, and bench indicate that when the initial candidate pool lacks poses capable of effectively observing difficult regions, subsequent viewpoint selection and optional-surface supplementation cannot readily compensate for the missing viewing directions. After observation-quality control was removed, the 25° effective coverage values of the four models were 68.76%, 82.52%, 73.04%, and 80.96%, respectively. These results show that the quality terms not only improve incidence-angle quality but also influence the coverage profile through candidate ranking under a limited viewpoint count.

Local pose replacement is primarily used to address residual regions remaining after the initial must-inspect-surface viewpoint selection. Under the fixed total viewpoint counts used in Table 4, removing this component reduced the must-inspect coverage of the regular table, long table, chair, and bench from 100.00%, 100.00%, 99.86%, and 98.16% to 99.90%, 99.02%, 99.12%, and 96.44%, respectively. Thus, local pose replacement improved the final must-inspect coverage by 0.10–1.72 percentage points without changing the total viewpoint count.

The stage-comparison procedure yielded must-inspect viewpoint counts of 14, 15, 22, and 55 for the regular table, long table, chair, and bench, respectively. The local pose replacement branch achieved must-inspect coverage rates of 100.00%, 100.00%, 99.86%, and 98.15%, respectively, which were no lower than the corresponding 99.996%, 99.459%, 99.131%, and 96.658% obtained by continued viewpoint addition. For the continued-viewpoint-addition branch, three decimal places are retained to distinguish small differences between the two branches.

These results verify the complementary roles of the three components. Candidate-pool iteration expands the set of effective candidates, observation-quality control improves coverage quality under a limited viewpoint count, and local pose replacement further reduces the remaining local coverage gaps without increasing the number of viewpoints.

### 5.4. Optional-Surface Supplementation Analysis

**Figure 8:**
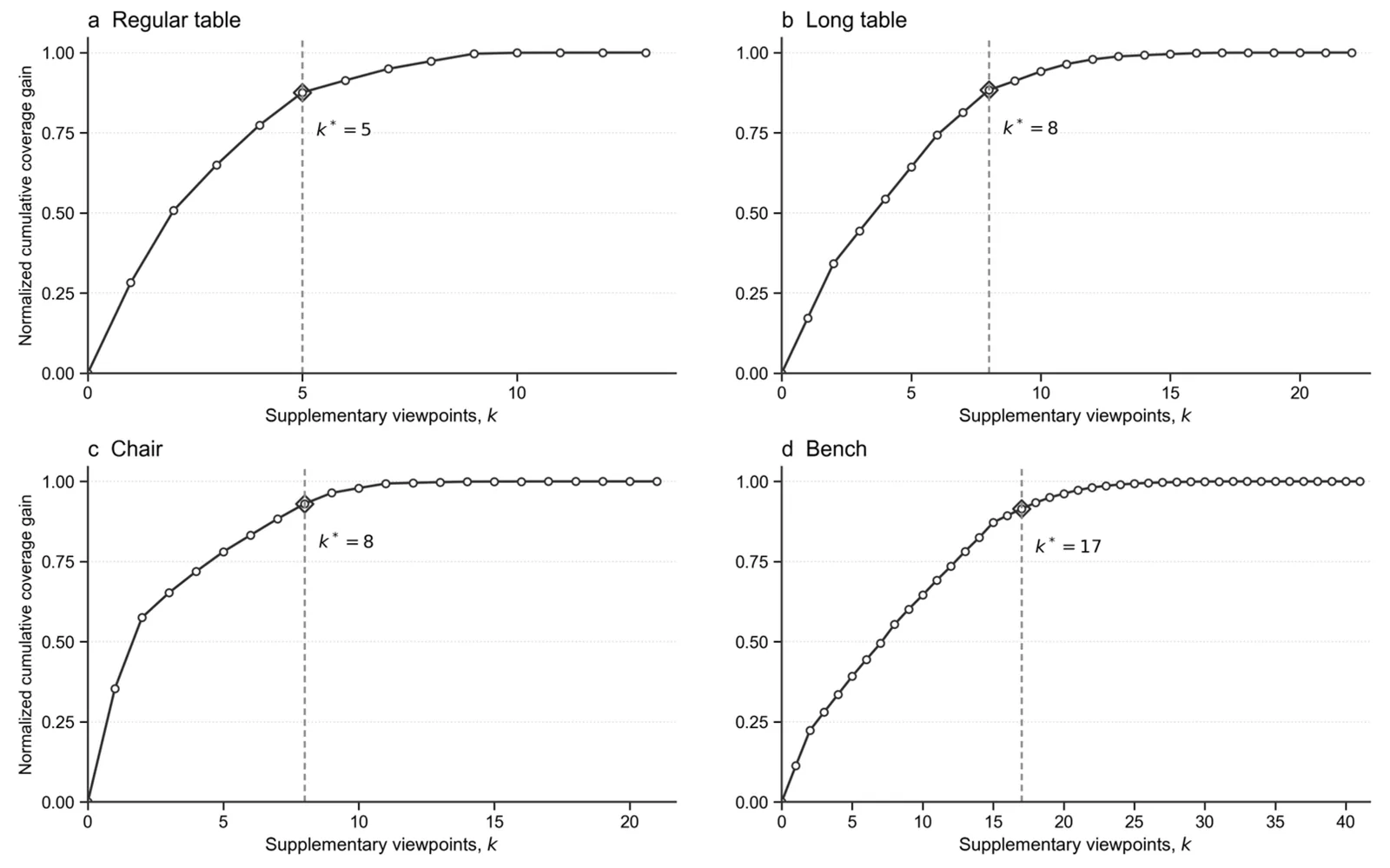
Cumulative optional-surface coverage gains and L-method knee points.

After local pose replacement was completed, the must-inspect-surface viewpoint set was fixed, and the complete positive-gain sequence of supplementary viewpoints was generated from the final candidate pool. Figure 8 shows the cumulative aggregate coverage-gain curves for the optional surfaces and the knee points determined using the L-method. The knee points for the regular table, long table, chair, and bench occurred at the fifth, eighth, eighth, and 17th supplementary viewpoints, respectively, yielding final total viewpoint counts of 19, 23, 30, and 72.

Before the knee points, the mean aggregate coverage gains for the four models were 35.00, 22.04, 31.73, and 15.80 percentage points per viewpoint, respectively. After the knee points, the corresponding mean gains decreased to 3.11, 1.67, 1.46, and 1.05 percentage points per viewpoint. All four models exhibited a clear decline in gain after the knee point. Although additional viewpoints could still increase optional-surface coverage, the marginal coverage gain provided by each additional viewpoint had become relatively small. For each model, the pre-knee mean is calculated from the per-viewpoint aggregate gains from the first supplementary viewpoint through the knee point, whereas the post-knee mean is calculated over the remaining positive-gain viewpoints after the knee point.

These results show that optional-surface supplementation exhibits a clear long-tail pattern. The L-method can therefore determine the number of supplementary viewpoints from the cumulative gain curve, retaining substantial optional-surface coverage gains while excluding the low-gain tail of the supplementation sequence.

### 5.5. Keep-Out-Zone-Constrained Visit-Sequencing Results

To evaluate the effect of the hard keep-out-zone constraint on visit sequencing, 20 paired trials were conducted for each model. The Euclidean-distance ACO+2-opt was compared with keep-out-zone-constrained ACO+2-opt using the same viewpoint sets, paired random seeds, and algorithm parameters.

**Figure 9:**
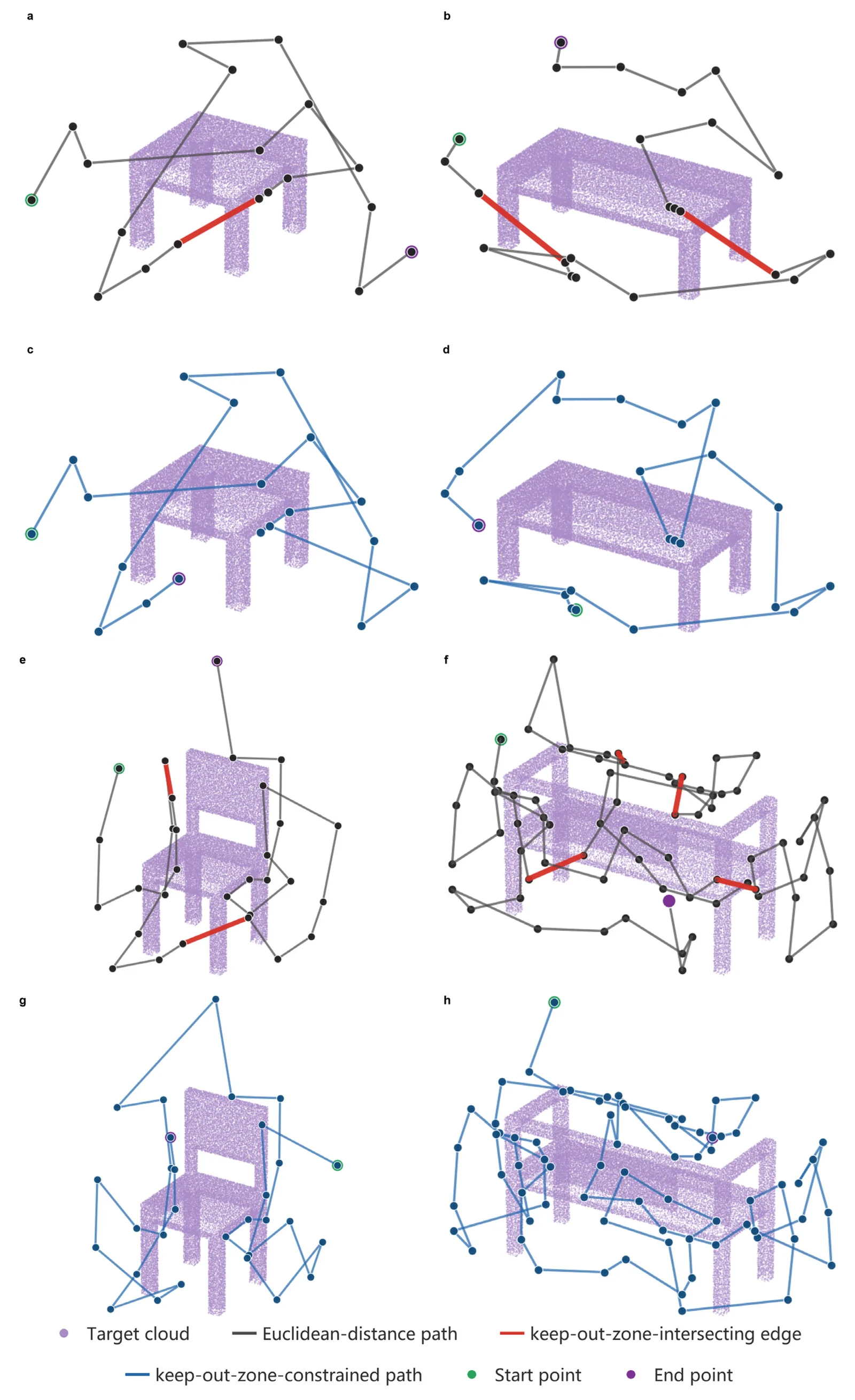
Comparison of Euclidean-distance and keep-out-zone-constrained viewpoint visit paths.

Table 5 presents the mean path lengths and mean numbers of keep-out-zone-intersecting edges over 20 runs for the four models, while Figure 9 compares the paths generated by the two methods. With Euclidean distance alone, the mean numbers of keep-out-zone-intersecting edges for the regular table, long table, chair, and bench were 1.00, 2.00, 3.05, and 4.40, respectively. Under the hard keep-out-zone constraint, no intersecting edge occurred in any of the 80 runs. Relative to the Euclidean-distance method, the corresponding mean path lengths increased by 3.97%, 1.33%, 6.91%, and 1.56%, respectively.

The paired statistical tests were consistent with the trends shown in Table 5. For all four models, the differences in both path length and the number of keep-out-zone-intersecting edges were statistically significant, with all Holm-adjusted *p*-values no greater than 1.51 × 10^−4^.

These results show that the keep-out-zone-constrained method maintained zero keep-out-zone-intersecting viewpoint connections across all 80 runs, with path-length increases limited to 1.33–6.91%. As an independent geometric check, none of the 80 keep-out-zone-constrained routes intersected the original furniture triangle meshes. The viewing directions of the final viewpoints are shown in Figure 5. Figure 9 focuses on the visit sequences and keep-out-zone-intersecting connections.

### 5.6. Sensitivity and Robustness Analysis

#### 5.6.1. Parameter Sensitivity

Parameter sensitivity was evaluated on the long-table model with a fixed candidate pool, target-point set, visibility matrix, and *K_m_* = 15. The projection-quality weights and must-inspect viewpoint-selection coefficients were perturbed one at a time by ±20%, with the projection-quality weights renormalized after perturbation. The local-replacement coefficients, *ε_C_*, *ε_θ_*, and *β*, were varied over the ranges listed in Table 6.

Across the tested parameter settings, the must-inspect viewpoint selection retained the same 15-view set, the local-replacement stage accepted the same two replacements, and the optional-surface stage retained the same supplementary-viewpoint sequence and knee point k^*^ = 8. The reported coverage metrics were unchanged, indicating stable planning outputs over the tested ranges.

#### 5.6.2. Viewpoint-Budget Robustness

To examine whether the fixed-budget comparison depends on the particular *K_m_* determined by the stage-branch comparison, two neighboring budgets were additionally tested for each model. The tested budgets were *K* = {12, 14, 16}, {13, 15, 17}, {20, 22, 24}, and {53, 55, 57} for the regular table, long table, chair, and bench, respectively. At each budget, the proposed selector, GA, and BPSO used the same frozen candidate pool, target-point set, visibility matrix, and exact viewpoint count; local pose replacement, optional-surface supplementation, and path planning were disabled. The proposed selector is deterministic, whereas the GA and BPSO values in Table 7 are means over 20 independent runs.

Across all 12 model-budget combinations, the proposed selector achieved higher *C_must_* and *C_25_* than the 20-run means of both GA and BPSO, indicating that the observed geometric-coverage and low-incidence-angle coverage advantages were not restricted to a single *K_m_* value.

### 5.7. Discussion and Limitations

The quality of the candidate space determines the upper bound on the coverage achievable through subsequent viewpoint selection. The ablation results show that removing candidate-pool iteration substantially reduces the coverage of the long table, chair, and bench, indicating that subsequent optimization cannot readily compensate for effective viewing directions that are absent from the candidate space. An excessively large candidate pool also introduces considerable computational cost. Taking the bench model as an example, the mean runtimes of GA, BPSO, and the proposed method were 186.57 ± 3.67 s, 115.07 ± 24.73 s, and 144.89 ± 0.68 s, respectively, indicating that the computational cost of the proposed method lies between those of the two stochastic optimization baselines. Iterative updating improves the effectiveness of the candidate pool by focusing computation on uncovered regions and regions with relatively low observation quality while controlling the candidate-pool size. In offline planning, the visibility matrix can also be precomputed and reused to avoid repeated visibility evaluation.

Geometric coverage alone does not fully characterize acquisition quality. The fixed-count comparisons show that substantial differences in low-incidence-angle effective coverage can remain even when the geometric coverage levels are similar. Viewpoint selection should therefore consider both geometric coverage and incidence-angle quality. The coverage and observation-quality metrics used in this study characterize front-end image-acquisition conditions and do not directly represent actual defect-detection accuracy. Actual defect-detection performance also depends on the downstream defect-recognition algorithm and practical imaging conditions.

The optional-surface experiments show that the marginal coverage gain per viewpoint gradually decreases as the number of supplementary viewpoints increases. The L-method determines the number of supplementary viewpoints from the cumulative gain curve, thereby avoiding excessive viewpoint addition during the low-gain stage of the supplementation sequence.

The path experiments further show that optimizing the visit sequence solely according to Euclidean distance may result in straight-line connections between adjacent viewpoints that intersect keep-out zones. The hard keep-out-zone constraint excludes such connections during both ACO construction and 2-opt refinement, with only a limited increase in path length. The magnitude of this increase depends jointly on the viewpoint distribution and the geometry of the keep-out zones.

This study focuses on offline geometric planning using known STL models. Future work will extend the framework to robot-arm kinematics, model-to-object and camera-calibration deviations, and real-world visual acquisition experiments to evaluate viewpoint reachability, motion execution, and defect-image acquisition performance.

## 6. Conclusions

For the visual inspection of panel furniture with known STL models, this study proposes a task-oriented multi-view acquisition planning method comprising inspection-surface prioritization, residual-driven candidate-pool iteration, two-stage viewpoint selection, and keep-out-zone-constrained visit sequencing. Surface coverage is quantified using area-weighted target points, while incidence angle and projection quality are also evaluated. Local pose replacement addresses residual coverage gaps on must-inspect surfaces, the L-method determines the number of supplementary viewpoints for optional surfaces, and keep-out-zone-constrained ACO+2-opt generates the visit sequence.

For the regular table, long table, chair, and bench, the complete method achieved must-inspect-surface coverage rates of 100.00%, 100.00%, 99.86%, and 98.16% using 19, 23, 30, and 72 viewpoints, respectively, with 35° effective coverage ranging from 97.80% to 99.86%. The comparative and ablation results show that quality-aware selection improves low-incidence-angle effective coverage under limited viewpoint counts, candidate-pool iteration substantially expands the attainable coverage of complex structures, and local pose replacement further reduces remaining coverage gaps without increasing the number of must-inspect-surface viewpoints. The L-method determined five, eight, eight, and 17 supplementary viewpoints for the optional surfaces, respectively. In the path experiments, keep-out-zone-constrained sequencing maintained zero intersecting edges for all four models, with path-length increases of 1.33–6.91%. These results show that the proposed method establishes a planning framework encompassing inspection-surface prioritization, candidate-viewpoint generation, viewpoint selection, and keep-out-zone-constrained visit sequencing, thereby providing methodological support for front-end multi-view acquisition in panel-furniture visual inspection.

## Figures and Tables

**Table 1:** Final viewpoint-planning results for four furniture models.

Model	Total Viewpoints	Must-Inspect-Surface Metrics					Optional-Surface Coverage		
		C_must_/%	C_25_/%	C_35_/%	Mean Angle/°	P_95_/°	Class 1/%	Class 2/%	Class 3/%
Regular table	19	100.00	83.05	99.86	16.29	29.51	89.98	85.03	—
Long table	23	100.00	91.00	99.83	16.35	27.16	85.79	90.50	—
Chair	30	99.86	73.80	99.84	19.20	30.35	73.76	96.56	100.00
Bench	72	98.16	90.05	97.80	12.21	27.32	86.60	89.15	98.97

**Table 2:** Deterministic-selector results at matched must-inspect-surface viewpoint counts.

Model	Method	Viewpoints	C_must_/%	C_25_/%	C_35_/%	Mean Angle/°	P_95_/°
Regular table	Our method	14	**99.90**	**80.96**	**99.01**	**16.68**	**29.86**
	Q-NBV		98.66	66.93	95.84	19.40	32.96
	GSC		99.39	73.53	97.40	18.10	31.59
Long table	Our method	15	**99.90**	**84.99**	**99.58**	**16.87**	**30.16**
	Q-NBV		98.42	38.08	59.11	33.60	61.53
	GSC		99.73	56.69	87.37	23.82	46.96
Chair	Our method	22	98.01	**73.24**	97.55	**18.79**	**30.95**
	Q-NBV		98.80	67.61	**97.95**	20.02	31.59
	GSC		**99.14**	66.62	97.14	20.48	32.35
Bench	Our method	55	96.31	**90.98**	**96.26**	**9.95**	**25.34**
	Q-NBV		96.11	79.88	91.02	14.33	36.82
	GSC		**97.12**	80.96	90.98	16.22	43.64

**Table 3:** Comparison with GA and BPSO at matched must-inspect-surface viewpoint counts.

Model	Method	Viewpoints	C_must_/%	C_25_/%	C_35_/%	Mean Angle/°	P_95_/°
Regular table	Our method	14	**99.90**	**80.96**	**99.01**	16.68	29.86
	GA		92.93 ± 2.18	79.78 ± 2.74	92.46 ± 2.18	**16.22 ± 0.50**	**29.12 ± 1.13**
	BPSO		76.02 ± 5.21	60.87 ± 2.62	74.41 ± 5.09	17.13 ± 1.27	31.57 ± 1.86
Long table	Our method	15	**99.90**	**84.99**	**99.58**	**16.87**	**30.16**
	GA		89.07 ± 3.43	71.83 ± 4.18	87.64 ± 3.72	17.99 ± 0.79	31.39 ± 1.07
	BPSO		68.67 ± 3.67	48.27 ± 3.78	63.54 ± 3.58	20.85 ± 0.79	39.47 ± 5.03
Chair	Our method	22	**98.01**	**73.24**	**97.55**	18.79	**30.95**
	GA		89.80 ± 3.10	68.49 ± 3.26	88.50 ± 3.38	**18.09 ± 0.47**	31.78 ± 1.37
	BPSO		68.00 ± 5.33	48.46 ± 2.70	64.94 ± 5.25	18.87 ± 0.88	34.50 ± 1.84
Bench	Our method	55	**96.31**	**90.98**	**96.26**	9.95	25.34
	GA		89.07 ± 1.41	85.18 ± 1.38	88.76 ± 1.40	**7.84 ± 0.68**	**24.56 ± 0.59**
	BPSO		69.77 ± 1.59	63.06 ± 1.48	68.22 ± 1.45	10.22 ± 0.94	29.11 ± 1.54

**Table 4:** Component-ablation results at fixed total viewpoint counts.

Model	Configuration	Viewpoints	C_must_/%	C_25_/%	C_35_/%
Regular table	Our method	19	**100.00**	**83.05**	**99.86**
	No candidate-pool iteration	19	97.68	79.09	97.12
	No local pose replacement	19	99.90	80.96	99.01
	No observation-quality control	19	95.31	68.76	93.13
	Our method	23	**100.00**	**91.00**	**99.83**
Long table					
	No candidate-pool iteration	23	78.25	39.44	64.15
	No local pose replacement	23	99.02	88.03	98.85
	No observation-quality control	23	**100.00**	82.52	99.66
Chair	Our method	30	**99.86**	**73.80**	**99.84**
	No candidate-pool iteration	30	51.25	28.43	36.87
	No local pose replacement	30	99.12	70.92	98.88
	No observation-quality control	30	98.52	73.04	98.15
Bench	Our method	72	**98.16**	90.05	**97.80**
	No candidate-pool iteration	72	60.02	59.41	59.91
	No local pose replacement	72	96.44	**90.98**	96.26
	No observation-quality control	72	97.22	80.96	90.98

**Table 5:** Paired visit-sequencing results for Euclidean-distance and keep-out-zone-constrained methods.

Model	Mean Path Length, Euclidean/mm	Mean Path Length, Keep-Out-Zone-Constrained/mm	Mean Path-Length Increase/%	Mean Intersecting Edges, Euclidean	Mean Intersecting Edges, Keep-Out-Zone-Constrained
Regular table	4100.53	4263.31	3.97	1.00	0.00
Long table	4820.03	4883.99	1.33	2.00	0.00
Chair	6317.11	6753.40	6.91	3.05	0.00
Bench	20,136.99	20,450.75	1.56	4.40	0.00

**Table 6:** Parameter-sensitivity settings and planning stability for the long-table model.

Component	Parameters	Tested Range	Result
Projection quality	Weights in Equation (6)	Each weight ±20%; renormalized	Same 15-view set
Must-inspect viewpoint selection	Coefficients in Equation (14)	Each coefficient ±20%	Same 15-view set
Local pose replacement	Coefficients in Equation (15), *ε_C_*, *ε_θ_*	Coefficients ±20%; *ε_C_* = {0, 0.01, 0.02, 0.05} pp; *ε_θ_* = {0, 0.50°, 0.75°, 1.00°}	Same two replacements
Optional-surface supplementation	*β*	*β* = {0.20, 0.35, 0.50}	Same supplementary sequence and knee point, k^*^ = 8

**Table 7:** Viewpoint-budget robustness analysis around *K_m_* for the four furniture models.

Model	K	Proposed		GA		BPSO	
		C_must_/%	C_25_/%	C_must_/%	C_25_/%	C_must_/%	C_25_/%
Regular table	12	**97.56**	**74.92**	89.96	74.54	72.12	56.06
	14	**99.90**	**80.96**	92.93	79.78	76.02	60.87
	16	**100.00**	**87.96**	95.58	84.93	80.71	64.24
Long table	13	**99.02**	**82.21**	85.34	66.46	66.17	46.98
	15	**99.90**	**84.99**	89.07	71.83	68.67	48.27
	17	**99.95**	**85.12**	91.87	76.22	69.58	49.64
Chair	20	**96.89**	**71.51**	87.48	67.60	66.68	48.11
	22	**98.01**	**73.24**	89.80	68.49	68.00	48.46
	24	**98.86**	**74.62**	90.87	70.15	68.11	48.26
Bench	53	**95.21**	**89.76**	88.27	84.34	69.16	62.66
	55	**96.31**	**90.98**	89.07	85.18	69.77	63.06
	57	**96.68**	**92.17**	89.85	86.58	70.38	63.84

## Data Availability

The original contributions presented in this study are included in the article. Further inquiries can be directed to the corresponding author.
